# Texture Analysis for the Bone Age Assessment from MRI Images of Adolescent Wrists in Boys

**DOI:** 10.3390/jcm12082762

**Published:** 2023-04-07

**Authors:** Rafal Obuchowicz, Karolina Nurzynska, Monika Pierzchala, Adam Piorkowski, Michal Strzelecki

**Affiliations:** 1 Department of Diagnostic Imaging, Jagiellonian University Medical College, 31-008 Krakow, Poland; rafalobuchowicz@su.krakow.pl; 2 Department of Algorithmics and Software, Silesian University of Technology, 44-100 Gliwice, Poland; 3 Selvita S.A., 30-348 Krakow, Poland; monika.pierzchala@selvita.com; 4 Department of Biocybernetics and Biomedical Engineering, AGH University of Science and Technology, 30-059 Krakow, Poland; pioro@agh.edu.pl; 5 Institute of Electronics, Lodz University of Technology, 93-590 Lodz, Poland; michal.strzelecki@p.lodz.pl

**Keywords:** bone age, image texture, bone MRI, pediatric radiology, regression analysis

## Abstract

Currently, bone age is assessed by X-rays. It enables the evaluation of the child’s development and is an important diagnostic factor. However, it is not sufficient to diagnose a specific disease because the diagnoses and prognoses may arise depending on how much the given case differs from the norms of bone age. Background: The use of magnetic resonance images (MRI) to assess the age of the patient would extend diagnostic possibilities. The bone age test could then become a routine screening test. Changing the method of determining the bone age would also prevent the patient from taking a dose of ionizing radiation, making the test less invasive. Methods: The regions of interest containing the wrist area and the epiphyses of the radius are marked on the magnetic resonance imaging of the non-dominant hand of boys aged 9 to 17 years. Textural features are computed for these regions, as it is assumed that the texture of the wrist image contains information about bone age. Results: The regression analysis revealed that there is a high correlation between the bone age of a patient and the MRI-derived textural features derived from MRI. For DICOM T1-weighted data, the best scores reached 0.94 R2, 0.46 RMSE, 0.21 MSE, and 0.33 MAE. Conclusions: The experiments performed have shown that using the MRI images gives reliable results in the assessment of bone age while not exposing the patient to ionizing radiation.

## 1. Introduction

Age is an imprinted parameter both from a medical and legal point of view [1,2]. There are many surgical and non-surgical procedures where precise estimation is very important [3]. In addition to medical issues, there are a wide range of non-medical subjects, e.g., legal problems and qualifications in competitive sports, where a precise age estimation is mandatory [4,5]. Legal issues become more important due to migration, especially in countries where birth records can be lost [6,7].

In the past century, it has emerged that the most accurate biological indicator of bone age is skeletal maturity. Bone age reflects the biological age of the patient, including hormonal and socioeconomic factors that are modulators of the growth and maturation of the child [8,9,10]. Therefore, this may be different from chronological age, especially in cases where factors that affect development are pushed to extremes, such as stress, malnutrition, or endocrine disorders [11]. With the development of radiological techniques, it has emerged that methods used for bone scanning could also be used for age determination. Therefore, X-ray-based techniques have emerged, exclusively in upper- and middle-class Caucasian populations [12,13], which nowadays gain criticism for their applicability due to racial and social differences [14,15,16,17,18]. With the advent of modern diagnostic techniques, there are attempts to use them in scanning for the estimation of the age of patients. Additionally, as a consequence, ultrasound, magnetic resonance (MR), and even computed tomography (CT) were employed [19,20,21,22,23]. The MR approach is of great interest because it is radiation-free and provides a detailed representation of tissues, including growth plates and nuclei [24,25,26]. The texture of such an image reflects the bone structure that is visualized in MR images. Textures represent complex patterns that are coded in the data and are built from points of different brightness and distribution. The distribution of the pixels and their characteristics can be analyzed by many textural features [27,28], such as phase frequency, coarseness, and regularity of randomness direction, to name a few [1]. Careful analysis of the initial textural pattern provides standardized feature extraction, which goes beyond the recognizable abilities of the human eye [29,30,31], allowing quantitative analysis of various medical images [32,33]. Changes in the growth zone and bone marrow composition reflect the maturation of the long bone as the site of dynamic morphological changes [34,35,36,37,38].

Since a bone age assessment is of great importance, this topic has been addressed in order to support physicians with an automated analysis of the data, making this task less labor intensive. In the literature, there are many approaches to address this problem, when analyzing X-ray images of hands [39,40,41,42,43,44,45], the chest [40,46], or whole-body images [47,48]. In the case of a fully automated deep learning approach, first the hand region was determined in the image using the U-Net network for semantic segmentation of the hand region, then the image registration was applied to allow for an easy determination of hand regions corresponding to each other between various images. Here, a deep learning approach for key point selection was also adopted. Finally, another network was used to solve the regression task and predict age. A similar pipeline was introduced in previous studies [40,44,45], yet the authors underlined the importance of transfer learning when preparing regression models. There were also approaches that used one network for the evaluation of bone age, as presented in the research in which whole-body scans were analyzed using well-known deep architectures, such as VGGNet, GoogLeNet, and ResNet, to find the best solution [47], or the hand X-ray image was analyzed with the attention-Xception network [43]. In [46] not only was the age determined from the chest radiograph images, but also, they analyzed the activation maps to find the most characteristic regions that influence the patient’s age. Instead of using regression models, generative adversarial networks (GANs) were exploited to decide bone age [42]. It was also possible to estimate the age from the bone mineral density at Ward’s triangle and the trabecular volume measured in the iliac crest [49,50]. We should not resign from more traditional approaches based on histogram thresholding, which allowed a precise determination of the chondorous part of the growth plate [51,52,53].

Most bone age assessment techniques implement X-ray-based imaging modalities that are invasive to some extent for patients. In this work, we would like to test whether other, non-invasive imaging techniques enable an accurate age estimation from acquired images that contain bone tissue. The aim of the present study was to explore whether the long bone textural analysis of the growth region on MRI images reflects changes in the age of the child and can possibly be applied for the determination of the bone age. To perform that examination, a dedicated database of MRI scans of adolescent hands was prepared. The descriptive region in the scan was marked manually, and then the textural features were extracted and the regression analysis was applied.

## 2. Materials and Methods

To verify whether it is possible to determine bone age from MRI images, a suitable dataset had to be prepared. The data acquisition is described in detail. The dataset gathered is briefly described. Next, the proposed textural features are presented, and details concerning the experiment quality measurements are given, followed by a description of experiment methodology.

### 2.1. Data Acquisition

This study was carried out according to the guidelines of good medical practice. The images were taken from a group of male volunteers, and the acquisition was approved by the Ethics Committee of Jagiellonian University (permission no. 1072.6120.16.2017) and complied with the Declaration of Helsinki. Written informed consent for participants was obtained from their legal guardians. The left hand of 30 healthy boys was examined by a 1.5 T system (GE Optima 360, Chicago, IL, USA) with a dedicated four-channel wrist coil. The acquisition was performed in a prone ‘superman’, e.g., with a hand erected in the overhead position.

Table 1 presents the parameters used to create T1-weighted and T2-weighted images. During the acquisition, 286 × 286 matrix size was used for the study. The scanning time of one sequence was in the range of 87–124 s. Radius growth plates were analyzed in coronal scans. Images were archived using the SIEMENS PACS (SYNGO, Siemens Healthineers, Erlangen, Germany). Anonymized studies were subsequently retrieved for image post-processing. The qMaZda software [54] was used for the computation of texture feature maps. Images with non-correctable motion artifacts were rejected. Small corrections of movement artifacts were performed with the use of pixel-by-pixel positioning of the overlaid images, and masks were developed that could compensate for the horizontal and vertical movements by a given number of pixels in case of minor movements.

### 2.2. Dataset Description

**Figure 1:**
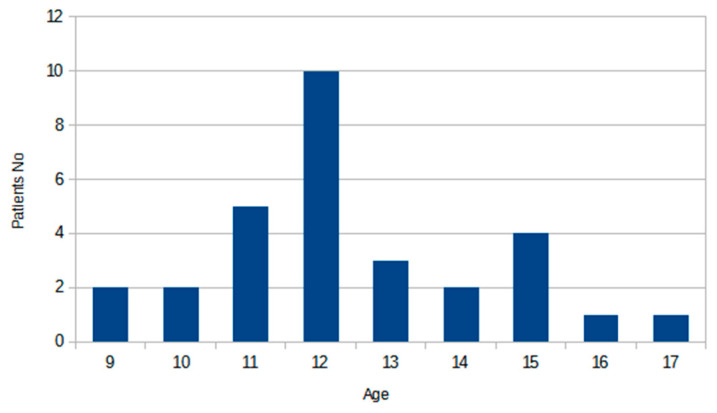
Distribution of the age of the patient within the dataset.

**Figure 2:**
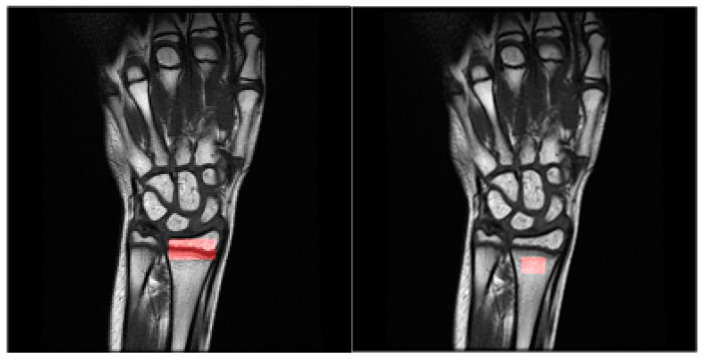
Exemplary magnetic resonance imaging. The red rectangle shows the growth region in the image on the right and bone region on the left scan.

The dataset consists of images that show the bone structure of the non-dominant hand (left in all cases analyzed). The subject’s ages ranged from 9 to 17, with an average age of 12.43 and a median age of 12. The detailed distribution of age within the dataset is presented in Figure 1. There were accessible original DICOM (Digital Imaging and Communications in Medicine) images, and the visual information was stored in 12 bits. Furthermore, these images were normalized to the 0–255 range and stored as an 8-bit PNG image. In both cases, the image resolution is 512 × 512 pixels. Figure 2 presents an example of such a scan. In total, there are 55 images recorded with T1-weighted MRI and the same number of scans are collected with T2-weighted MRI. In image acquisition, we used a coronal plane of 3 mm thick, where a maximum of 10 layers were used in the plane with radius. From these data, up to three images per patient were considered usable considering their quality and visibility of the region of interest. In the case of the bone analysis, we used all 55 images; however, for radius growth region, only 30 images were used. The pixel spacing of the recorded data varies from 0.2539 to 0.3516 pixels, where the largest group of images was obtained for a 0.293 pixel spacing.

### 2.3. Data Analysis Methods and Methodology

The starting point of the data analysis was to select a descriptive region of interest (ROI), which well characterizes bone structure and allows for calculating the textural features, which become the mathematical description of the data. Since the pixel spacing of the data differs, we have decided to test two approaches. Firstly, a constant ROI size was selected in the MRI of the forearm. Second, the ROI size differed assuring the same metric units. These regions were applied to the DICOM and PNG versions of the data. Moreover, two regions with different medical meanings were considered: the bone and the growth region; please refer to Figure 2 for visualization.

For each region, the textural features were calculated using the qMaZda software. The software supports rich functionality, from which we have chosen several options. At the beginning, ±3σ normalization of the input region *I*(*x*,*y*) was applied. It is beneficial when the image histogram is close to the Gaussian, and it was also proved that better results are obtained for MR data. For an image, the mean *μ* and standard deviation σ of illuminance were calculated. Then, the image was scaled by recalculating min*_norm_ = μ* − 3*σ* and max*_norm_ = μ* + 3*σ*, and finally, thresholded, according to Equations (1) and (2):

(1) $$N\left(x,y\right)\;=\;\frac{I\left(x,y\right)-\underset{\mathrm{norm}}{\min}}{\underset{\mathrm{norm}}{\max}\;-\;{\underset{\mathrm{norm}}{\min}}^{\prime }}$$

(2) $$I_{norm}\left(x,y\right)=\left\{\begin{array}{cc} 255 & N\left(x,y\right)>255\\ N(x,y) & 0\leq N(x,y)\leq 255\\ 0 & N\left(x,y\right)<0 \end{array}\right.$$

Then, for the region, the textural features were calculated. Starting from the first-order features that describe illumination distribution within the histogram, by 9 parameters: mean, variance, skewness, kurtosis, and percentiles 1, 10, 50, 90, and 99. The spatial relations between pixels were also exploited to derive features from the gray-level co-occurrence matrix (GLCM) [27], run-length matrix (RLM) [55], gradient matrix [27], first-order autoregressive model (AR) [56], Haar wavelet transform (HW) [57], Gabor transforms, and histogram of oriented gradients (HOG) [58]. There are 11 parameters calculated from the GLCM matrix in four spatial directions: horizontal, vertical, and two diagonals. This method is additionally parametrized with a distance between pixels treated as neighbors. In our research, this distance was set in a range from 1 to 5 pixels. Taking into account all those combinations (11 × 4 × 5), 220 features were obtained. From the RLM method, 20 additional parameters were calculated. This method gives five features, and they were calculated using the same four directions as in the GLCM method. There were five gradient matrices built with a high-pass filter using a 3 × 3 pixel mask. There were five features derived from the AR method. Their idea is based on the finding that brightness depends on the weighted sum of the neighboring pixels. The HW transform brings 16 parameters, which result from four down-sampled sub-images representing the energy of the data after conversion to the wavelet transform. In the case of the Gabor filter, the transformation was calculated in four directions (as for GLCM) using six Gaussian envelopes of the following sizes: 4, 6, 8, 12, 16, and 24. That gives 24 parameters. Finally, an eight-bin histogram of occurrences of gradient orientation in the image was calculated as a feature of the HOG method. After all, those transformations’ 307 features were obtained. The details on how to calculate each of these features are given in Appendix A.

Since the number of calculated features was large compared to the number of training samples, the reduction in feature space was necessary to remove redundancies and highly correlated data and improve the model’s possibility to derive patterns from the data. This was achieved by using principal component analysis (PCA). Finally, the bilayered perceptron neural network implemented in the Matlab Regression Learner toolbox was applied to perform the analysis. Since the datasets are small, the leave-one-out (LOO) cross-validation schema was used to validate the neural network. This means that the network was trained for all data samples except one. The remaining sample was used for validation. This process was repeated for all samples in the dataset, so that each sample was validated separately. In the LOO approach, the bias associated with the random selection of data for folds (as in the case of cross-validation) or with the selection of the test set (as in the case of train-test split) is reduced. Furthermore, the performance of the model can be assessed for each sample. The LOO method is recommended and is quite commonly used in the case of small datasets (from a dozen to several dozen samples) [59,60,61,62,63].

The quality regression model was evaluated by mean square error (MSE), root mean square error (RMSE), mean absolute error (MAE), and coefficient of determination (R2).

### 2.4. Experiment Setup

In the experiments performed, images of bone and growth regions have been analyzed. In the case of bones, all 55 images were used. However, for the growth region analysis, each patient was represented by only one image, which reduced the number of samples to 30. In this research, we have focused on the metric version of the annotation. Since the textural features depend strongly on the pixel relations, varying sizes of pixels might influence the outcomes, whereas when we had each time data with similar pixel spacing, this problem and its influence on results could be neglected. We performed a regression analysis regarding the patient’s full age. However, we noticed that the correlation improved when comparing texture features with the age given in months. Although that was an interesting finding, because of the small number of samples, it was abandoned for further investigation. The image preprocessing with the qMaZda software generated many texture features (307) to describe each sample. Using so many features can decrease regressor capabilities when most of them may not be correlated with the patient’s age. Therefore, a Spearman correlation between textural features and outcomes was calculated, which helped us choose 15 highly correlated, representative features. This procedure was applied to the training data separately for each model. Furthermore, as proven by previous research, these indicated the best textural features [64]. For this set of data, PCA was applied to derive the most discriminative parameters and remove any existing correlation between them. For bone and growth region analysis, the first 3 PCA eigenvectors were selected. The assumed number of PCA features used in regression analysis reflected the number of samples analyzed (55 in the case of bone image experiments and 30 when the growth region was evaluated). Experiments were performed for all four datasets: DICOM T1-weighted, DICOM T2-weighted, PNG T1-weighted, and PNG T2-weighted images. Furthermore, two tasks were evaluated: (1) finding the relation between the textural features of the bone region and the age of the patient; and (2) evaluating the dependence of the textural features in the growth region on the age of the patient.

## 3. Results

**Figure 3:**
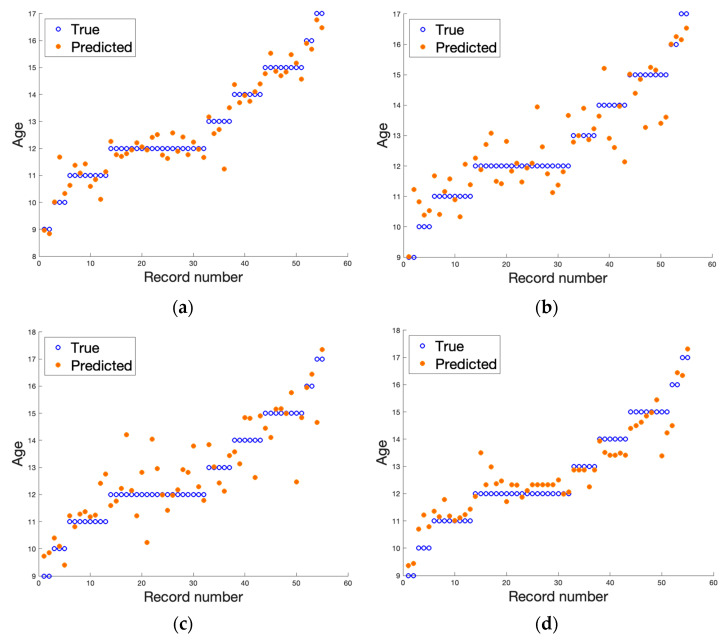
The result of regression algorithms for all cases examined for data describing bone images (metric). The real age is presented by blue circles and estimated age with a regression algorithm is depicted by orange dots. The samples are ordered on the y axis with increasing age. (**a**) DICOM T1-weighted, (**b**) DICOM T2-weighted, (**c**) PNG T1-weighted, and (**d**) PNG T2-weighted.

**Figure 4:**
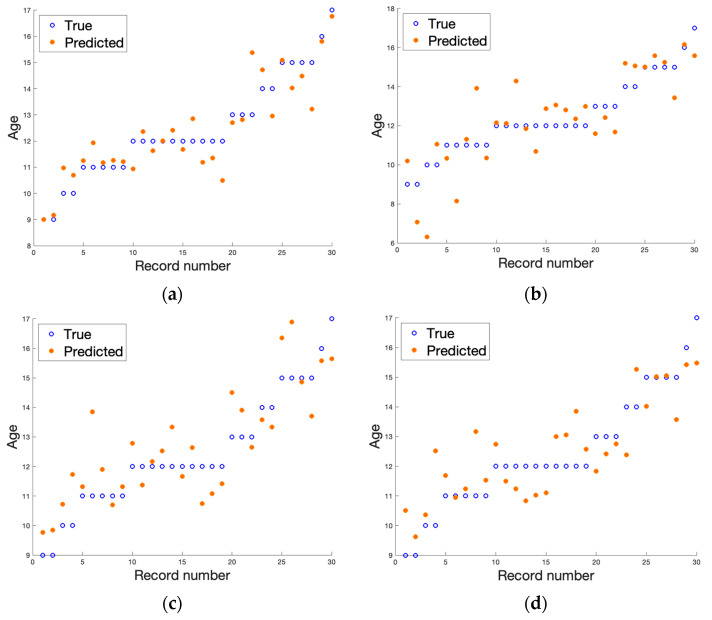
The result of regression algorithms for all examined cases for data describing growth region images (metric). The real age is given as a blue circle and age estimated with a regression algorithm is depicted as orange dot. The samples are ordered on the y axis with increasing age. (**a**) DICOM T1-weighted, (**b**) DICOM T2-weighted, (**c**) PNG T1-weighted, and (**d**) PNG T2-weighted.

Table 2 and Table 3 collect the best results obtained after applying a two-layer neural network from the Matlab Regression Learner toolbox. The visualization of the results is depicted in Figure 3 and Figure 4. The results presented in Table 2 and Table 3 are the average values of the regression errors obtained for each of the samples. The plots summarize the results of all the LOO trials. From the results presented, we can see that the regression analysis in all cases allowed the prediction of the age of a patient based on the textural features analysis of the MRI data. The gathered results suggest that, considering the bone region, we achieved more stable results with fewer errors. However, due to the limited amount of data, these discrepancies between the analyzed regions could be due to decreasing the number of samples from 55 (for the bone) to 30 (for the growth region). The dispersion coefficient is very high, yet significantly better results were obtained when a T1-weighted image using the original 12-bit DICOM data was considered. We achieved the best score, which was R2 equal to 0.94 for the original 12-bit representation (DICOM) when the bone region was evaluated. In this scenario, second place was taken by the T2-weighted image with a reduced number of bits to eight (R2 equals 0.87). When analyzing the results obtained for the growth region (see Table 3), again, the T1-weighted image in 12-bit format returns the best outcomes, yet other results deteriorate significantly. This finding was also reflected in the other error metrics showing the smallest error in the case of DICOM T1-weighted datasets (see bold font in Table 2 and Table 3). The slight difference in the quality of age prediction by the chosen regressors was also noticeable in the plots presented in Figure 3 and Figure 4. Here, the predictions go through all observations in the case of analyzing textural features from bone DICOM T1-weighted images (see Figure 3a) and are very close to this line when the growth region DICOM T1-weighted images are considered (see Figure 4a). Since the true age was rounded to an integer value, the small discrepancies should not be surprising, as in reality, patients had a different number of months. As we noticed previously, the data reflected better when a shorter period was considered.

## 4. Discussion

Radiographic techniques are well-established methods that are used for the determination of bone age [12,13]. There are techniques that are based not only on the wrist estimation but also on other parts of the skeleton, including the clavicle [65], elbow [66], pelvis [67,68], humerus [69], or calcaneus [70,71]. Dental studies become a focus as body parts are used for age estimation [72,73,74].

Moreover, with the development of computer hardware, different techniques were proposed to obtain information from the image, allowing for the creation of efficient age evaluation systems, for example, Shorthand and BoneXpert to name a few [75,76]. There are many modern techniques that are based on shape extraction algorithms with comparative techniques, including those based on artificial intelligence [77,78,79,80]. However, many of these methods are still based on an X-ray analysis, where X-ray dose issues cannot be omitted.

Age determination based on a single radiograph is associated with low doses [81]. However, a cumulative dose in cases where multiple X-rays must be performed might not be acceptable. Ultrasonography, however, which is free from radiation and easy to use, is known as an operator-dependent method, which is a serious drawback of this otherwise useful technique [82].

MR was proposed as a method free of radiation exposure, but it is also repetitive, and in this regard, according to the results, it is stable. Table 4 provided a comparison of the MAE metric for our solution and other approaches working on X-ray data. As we can see, it outperformed other methods markedly. A certain drawback of MRIs is the time needed for the exam, which forces cooperation with young patients. Regarding the success of the MR examination, parental assistance is very important [24]. Child safety and comfort was assured in this study. That was very important as unintentional movement caused by inconvenient body alignment disturbs image creation. This is especially important in a proposed method where a small region of interest in the growth plate is used; therefore, a perfect image is key to the success of the proposed solution. Child cooperation is mandatory. However as described by Terada et al. [23] and Dvorak et al [22], short exam time was sufficient condition to ensure the creation of proper images. In our study, in contradiction to the protocol proposed by Dvorak et al. [22], Hojreh et al. [26], Stern et al. [83], and Quasim et al. [84], in addition to the T1-weighted spin echo and the gradient echo sequence (as applied in [23,24]), a T2-weighted spin echo was used. The choice of a T2-weighted sequence was dictated by the need to discriminate the number of watery progenitor cells because the amount of signals from these watery compounds was used as an indicator of the immaturity of the growth zone. The dependence of the growth zone composition on age with possible detection with the MR technique was described in experimental and clinical studies by Ecklund et al. [85], who described the dependence of the signal of the growth region composition. This agrees with histological studies proposed by Ballock et al. [86] and Breur et al. [87], who precisely described the basis of the known fact that the pattern of ongoing calcification of the growth region reflects maturation with age, which is at the core of the signal changes that are analyzed in our study as one of the discriminators of long bone maturation. In a study by Yun et al. [88], the dependence between the MR growth plate signal assessed in MR and skeletal maturation was not presented; however, the authors performed a study in the younger children group. One must remember that the proposed analysis of the growth plate is based on the narrow tissue element of progenitor cells, which is less than 3 mm and is a niche compared to the surrounding bone [89,90].

Despite the relatively low volume of the growth region in the composition of highly watery cells, it is highly detectable by high MR sequences, which in the image analysis, were reflected by a high correlation with histogram parameters (a sensitive indicator of brightness distribution but not necessarily structure) and supported by the presented regression analysis. This is logical because, in the zone of highly watery progenitor cells, the defined structure is very sparse, but the signal is strong. It can be observed that the younger the patient (with a wider growth region and more fluid), the brighter the signal. In older children, the amount of watery progenitor cells was reduced at the expense of the calcified bone rim, with a subsequent reduction in the influence of the bright area.

In a comparison of the T1-weighted and T2-weighted sequences, regression occurred more accurately for T1-weighted images than for T2-weighted signals, which is at least in part due to a high tissue contrast created between less hydrated trabeculae due to hydroxyapatite and therefore, low signal bone elements and high signal bone marrow [37,38]. The discriminative effect of the T2-weighted image due to the good differentiation between the unconverted bone marrow and the trabeculae can be partially spoiled by shift artifacts due to chemical composition, but also by the thickness of the trabeculae [91,92].

A slight influence on the results was observed regarding the type of encoding, the DICOM format was better with the T1-weighted sequence, which might be due to the overall contrast in the image where the T1-weighted sequence sensitive to water produces high signal differentiation in the image that is associated with a significant amount of blood morphotic elements in the immature marrow. This observation is consistent with clinical observations where sequences with a high TR time are used to differentiate lesions due to high visual contrast [93]. It is also worth mentioning that comparing different MRI sequences is problematic; however, there are works showing that the registration of two series of MRI data is possible to some extent [94].

Recently, new algorithms for automated bone marrow segmentation from MRI data have been developed [95,96]. We are going to implement such algorithms in our future research, especially when a large image database will be collected. The challenge will be to modify these algorithms in such a way as to select a specific ROI from the segmented, whole bone marrow. We know that radiomic features are prone to many problems. One of them is the low repeatability of texture features when multicenter studies are performed. On the other hand, such studies are essential to ensure the reliable validation of the developed machine learning models. It was shown in [97] that normalization applied to muscle tissue images acquired by different MR scanners improved the reproducibility of the calculated selected texture features. We will further investigate the influence of various ROI normalization schemes’ texture feature repeatability and reproducibility. Another factor that affects the calculation of bone marrow radiomic features is the variation of signal intensity between different scanners [98] as well as its dependence on signal blur phenomena. It is mostly caused by a chemical shift and magnetic susceptibility artifact, which belong to a class of tissue-specific artifacts. Other, less important might be also geometric artifacts that come from tissue tilt. Bone marrow analysis is always challenging and requires optimal image acquisition and compensation of acquisition artifacts.

There are some limitations to this study. First, the image acquisition was relatively long. However, the scanning time was successfully overcome by the cooperative and motivated children. Since it was a pilot study and we only wanted to verify the hypothesis that it is possible to determine the bone age with high accuracy from the MRI images, a small group of patients was examined. For further studies, it should be extended. Finally, the error in the segmentation of the growth plate must be considered, given the small area of interest and the averaging effect due to the influence of the surrounding tissues.

## 5. Conclusions

This preliminary study presented the feasibility of implementing MRI bone scans in the bone age estimation protocols as a novel approach based on an analysis of the internal bone structure based on tissue texture. This is a different approach in comparison to shape and volume analyses based on X-ray techniques that are used today. To verify the hypothesis that it is possible to estimate the patient’s age based on an MRI hand scan, a database of non-dominant hands of 30 children was acquired. On those scans, the regions of interest were manually selected, distinguishing the bone and growth regions, both annotated with a constant pixel spacing. For these regions, textural features were calculated using the qMaZda software. To select the most representative data, a correlation analysis was performed followed by the PCA transform. Then, regression analysis was applied, which revealed a high correlation between the DICOM T1 images and the age of the patients (for the remaining types of analyzed data, the correlation was moderate). This is a significant finding, as it supports the claim that the use of the radiation-free technique can replace the current protocol based on X-ray scans. Moreover, we see that the magnetic resonance images convey complete information about bone structure and can be used interchangeably with X-ray images. The presented approach that implements machine learning on textures visualized in MRI scans is a novel solution toward the most complete analysis of bone age information, allowing for an accurate assessment of the biological age of the patient.

## Figures and Tables

**Table 1:** Parameters used for acquisition of T1- and T2-weighted images.

Parameter	T1-Weighted	T2-Weighted
Slice thickness	3 mm	3 mm
Repetition Time	435 ms	2749 ms
Echo Time	16 ms	106 ms
Number of averages	2	2
Spacing	3.5 mm	3.5 mm
Echo train length	23	23
Bandwidth	81 MHz	97 MHz

**Table 2:** Quantitative regression parameters for different types of data and bone images (metric). Results for 3 features derived from 15 by applying principal component analysis.

Data	R^2^	RMSE	MSE	MAE
DICOM T1-weighted	0.9383	0.4584	0.2101	0.3300
DICOM T2-weighted	0.7510	0.8365	0.6997	0.6229
PNG T1-weighted	0.7283	0.9344	0.8732	0.6922
PNG T2-weighted	0.8711	0.5731	0.3284	0.4429

**Table 3:** Quantitative regression parameters for different types of data and growth region images (metric). Results for 3 features derived from 15 by applying principal component analysis.

Data	R^2^	RMSE	MSE	MAE
DICOM T1-weighted	0.8041	0.8194	0.6714	0.6142
DICOM T2-weighted	0.6621	1.4104	1.9892	1.0969
PNG T1-weighted	0.6743	1.0550	1.1130	0.8740
PNG T2-weighted	0.5216	1.1058	1.2228	0.9228

**Table 4:** Results of performance of the presented method and other solutions for the estimation of bone age based on hand.

References	MAE (Months)
Liu et al. [42]	6.01
Iglovikov et al. [39]	6.10
Salim and Hamza [40]	6.38
Zulkifley et al. [43]	7.70
	**MAE (years)**
Nguyen et al. [47]	4.856
Our method	0.330 (T1 DICOM), 0.692 (T1 PNG)

## Data Availability

Data is unavailable due to privacy.

## Appendix A

Here, the following points provide detailed information about the definition of the texture feature parameters that were used in presented research.

1. First-order features

First-order features are calculated for a gray-scale image using formulae presented in Table A1. Here, *h* stands for the normalized histogram function of occurrences at the *G* pixel level for image of width *W* and height *H*.

**Table A1:** Formulae to calculate first order features from the gray-scale image.

Feature Name	Formulae	
Mean	$\mu =\frac{1}{W\cdot H}\sum_{x=0}^{W-1}\sum_{y=0}^{H-1}I\left(x,y\right)=\sum_{i=0}^{G-1}i\cdot h(i)$	(A1)
Variance	$\sigma^{2}=\frac{1}{W\cdot H}\sum_{x=0}^{W-1}\sum_{y=0}^{H-1}{\left(I\left(x,y\right)-\mu \right)}^{2}=\sum_{i=0}^{G-1}{\left(i-\mu \right)}^{2}\cdot h(i)$	(A2)
Skewness	$\sigma^{-3}\sum_{i=0}^{G-1}{\left(i-\mu \right)}^{3}\cdot h(i)$	(A3)
Kurtosis	$\sigma^{-4}\sum_{i=0}^{G-1}{\left(i-\mu \right)}^{4}\cdot h\left(i\right)-3$	(A4)
Percentile	$\sum_{i=0}^{G-1}h\left(i\right)\geq percentile$	(A5)

2. Gray-level co-occurrence matrix [27]

Table A2 presents the features derived from the gray-level co-occurrence matrix *p*. This matrix saves the information of the number of existing co-occurrence of pixels of intensities indexing this cells within an image in cells. It is calculated separately in four directions (horizontal, vertical, and two diagonals). It is also possible to parametrize the distance d between pixels considered as adjoining in our research *d* = 1…5. The *G* stands for the pixel gray-level values.

**Table A2:** Formulae to calculate gray level co-occurrence matrix from the gray-scale image.

Feature Name	Formulae	
Contrast	$\sum_{i=0}^{G-1}\sum_{j=0}^{G-1}{\left(i-j\right)}^{2}p(i,j)$	(A6)
Correlation	$\sum_{i=0}^{G-1}\sum_{j=0}^{G-1}\frac{ijp\left(i,j\right)-{µ}_{i}{µ}_{j}}{\delta_{i}\delta_{j}}$ where *µ_i_* is average and *δ_i_* is standard deviation for *p_i_*	(A7)
Homogeneity	$\sum_{i=0}^{G-1}\sum_{j=0}^{G-1}\frac{p(i,j)}{{1+\left(i-j\right)}^{2}}$	(A8)
Entropy	$-\sum_{i=0}^{G-1}\sum_{j=0}^{G-1}p\left(i,j\right){log}_{2}(p\left(i,j\right))$	(A9)
Angular second moment	$\sum_{i=0}^{G-1}\sum_{j=0}^{G-1}p^{2}\left(i,j\right)$	(A10)
Sum average	$\sum_{m=1}^{G}mp_{sum}\left(m\right)$	(A11)
Sum variance	$\sum_{m=1}^{G}{\left(m-sum-avg\right)}^{2}p_{sum}\left(m\right)$	(A12)
Difference variance	$\sum_{m=1}^{G}{\left(i-{µ}_{dif}\right)}^{2}p_{dif}\left(m\right)$	(A13)
Difference entropy	$-\sum_{m=1}^{G}p_{dif}\left(m\right)\log\left(p_{sum}\left(m\right)\right)$	(A14)
Sum entropy	$-\sum_{m=1}^{G}p_{sum}\left(m\right)\log\left(p_{sum}\left(m\right)\right)$	(A15)
Sum of squares	$\sum_{i=0}^{G-1}\sum_{j=0}^{G-1}{\left(i-µ\right)}^{2}p(i,j)$	(A16)

3. Run-length matrix [55]

The run-length matrix *p* stores information about the length of pixel runs having the same pixel value. Thus, here, each matrix cell is indexed by the pixel gray-level value *G*, and the length *L*. The total number of recorded runs is denoted by *n_r_*. Features derived from the run-length matrix are given in Table A3.

**Table A3:** Formulae to calculate run-length matrix features from the gray-scale image.

Feature Name	Formulae	
Short run emphasis	$\frac{1}{n_{r}}\sum_{i=0}^{G-1}\sum_{j=1}^{L}\frac{p(i,j)}{j^{2}}$	(A17)
Long run emphasis	$\frac{1}{n_{r}}\sum_{i=0}^{G-1}\sum_{j=1}^{L}p(i,j)\cdot j^{2}$	(A18)
Gray-level non-uniformity	$\frac{1}{n_{r}}\sum_{i=0}^{G-1}\left(\sum_{j=1}^{L}p(i,j)\right)$	(A19)
Run-length non-uniformity	$\frac{1}{n_{r}}\sum_{j=1}^{L}\left(\sum_{i=0}^{G-1}p(i,j)\right)$	(A20)
Run percent	$\frac{n_{r}}{W\cdot H}$	(A21)

4. Gradient matrix [27]

The gradient magnitude for image region of interest *I* is calculated as follows:

(A22) $$\left|Ɗ\right|=\sqrt{{\left(I\left(x,y+1\right)-I(x,y-1)\right)}^{2}+{\left(I\left(x+1,y\right)-I(x-1,y)\right)}^{2}}$$

and within this region the mean, variance, skewness, and kurtosis are calculated (see formulas already presented for first-order features in Table A1) and additionally, the non-zeros parameter is computed as a ratio of |Ɗ| > 0 to all pixels in region of interest.

5. First order autoregressive model [56]

The autoregressive model with four θ parameters is given with formulae:

(A23) $$I\left(x,y\right)=\theta_{1}\left(I\left(x-1,y\right)-µ\right)+\theta_{2}\left(I\left(x,y-1\right)-µ\right)+\theta_{3}\left(I\left(x-1,y-1\right)-µ\right)+\theta_{4}\left(I\left(x+1,y-1\right)-µ\right)+µ+e\left(x,y\right)$$

where

(A24) $$µ=\frac{\sum_{x,y\in ROI}I\left(x,y\right)}{\sum_{x,y\in ROI}1}$$

and *e* stands for error.

6. Haar wavelet transform [57]

A Haar discrete wavelet transform with its energies and frequencies is applied to four sub-band images returning the energy and three low-pass (L) and high-pass (H) filters in horizontal and vertical directions: HH, LH, and HL.

7. Gabor transform

The Gabor transform locally decomposes the image and is calculated separately in four directions (horizontal, vertical, and both diagonals). The frequency components are computed by convolution with a kernel:

(A25) $$k\left(x,y\right)=e^{\frac{-(x^{2}+y^{2})}{2\delta^{2}}}\left(cos\left(\beta \right)+jsin\left(\beta \right)\right)\mathrm{where}\;\beta =\omega \left(x\cos\alpha +y\sin\alpha \right)$$

where the parameters define the frequency *ϖ*, orientation *α*, and standard deviation of the Gaussian envelope *δ*. The average magnitude is calculated as follows:

(A26) $$Mag=\frac{\sum_{x,y\in ROI^{\prime }}k\left(x,y\right)}{\sum_{x,y\in ROI^{\prime }}1}$$

and the ROI’ is morphologically eroded.

8. Histogram of oriented gradients [58]

Histogram of oriented gradients divides the image into 8 × 8 pixels cells, where the gradient’s magnitudes and directions are calculated. This information is coded into an 8-bin histogram, where the bins reflect the quantized gradient directions.
